# ALIGNMENT OF COMMUNICATION AND SUPPORT NEEDS BETWEEN LGBTQ+ FAMILY CAREGIVERS OF HOSPICE PATIENTS AND PROVIDERS

**DOI:** 10.1093/geroni/igac059.322

**Published:** 2022-12-20

**Authors:** Kristin Cloyes, Maija Reblin, Megan Thomas Hebdon, Miranda Jones, Lee Ellington

**Affiliations:** Oregon Health & Science University, Portland, Oregon, United States; University of Vermont, Burlington, Vermont, United States; University of Texas at Austin, Austin, Texas, United States; University of Michigan Department of Psychology, Ann Arbor, Michigan, United States; University of Utah and Huntsman Cancer Institute, Salt Lake City, Utah, United States

## Abstract

Effective and equitable end-of-life care requires alignment between the support and communication needs of hospice family caregivers and the knowledge and competence of hospice care team members. We investigate alignment between LGBTQ+ caregivers and hospice providers in a secondary analysis of data collected in three studies: 1) Surveys identifying predictors of hospice providers’ (N=122) attitudes toward LGBTQ+ caregivers and patients; 2) focus groups assessing hospice interdisciplinary care team members’ (N=48) knowledge, training, and opinions regarding LGBTQ+ family caregiver and patient communication and care needs; and 3) in-depth interviews with LGBTQ+ hospice family caregivers (N=20). Synthesis of key findings show how alignment is impacted by lack of systematic, inclusive orientation and gender data, provider religiosity, expectations of disclosure, cisheteronormative assumptions about the universality of EOL experiences, and caregivers’ experiences of minority stress. We discuss implications for developing interventions to improve provider communication, empowering caregivers, and addressing hospice-specific sources of minority stress.

